# Improved Non-Piezoelectric Electric Properties Based on La Modulated Ferroelectric-Ergodic Relaxor Transition in (Bi_0.5_Na_0.5_)TiO_3_-Ba(Ti, Zr)O_3_ Ceramics

**DOI:** 10.3390/ma14216666

**Published:** 2021-11-05

**Authors:** Xingru Zhang, Yinan Xiao, Beining Du, Yueming Li, Yuandong Wu, Liyuan Sheng, Wenchang Tan

**Affiliations:** 1 Shenzhen Institute, Peking University, Shenzhen 518057, China; zxr_hit@126.com (X.Z.); ynxiao_pkusz@yeah.net (Y.X.); bndu10s@alum.imr.ac.cn (B.D.); ymli11s@alum.imr.ac.cn (Y.L.); wuy@ier.org.cn (Y.W.); twc@pkusz.edu.cn (W.T.); 2 PKU-HKUST Shenzhen-Hong Kong Institution, Shenzhen 518057, China

**Keywords:** BNT-based lead-free ceramics, electric properties, ferroelectric to ergodic relaxor state transition, La doping

## Abstract

The characteristic transition from ferroelectric (FE) to ergodic relaxor (ER) state in (Bi_0.5_Na_0.5_)TiO_3_ (BNT) based lead-free ceramics provides an efficient approach to bring a highly ordered phase back to a disordered one. It would be rational to utilize this transition to improve relevant non-piezoelectric properties based on domain decomposition. In this work, different La contents were introduced to 0.93(Bi_0.5_Na_0.5_)TiO_3_-0.07Ba(Ti_0.945_Zr_0.055_)O_3_ ceramics (BNT-BZT-*x*La) to induce evolution of ergodic degree. The results reveal that with increasing La content, both the FE-ER transition temperature *T*_F-R_ and depolarization temperature *T*_d_ shift towards room temperature, implying a deepened ergodic degree. By modulation of ergodic degree, thermally stimulated depolarization current experiment shows a higher current density peak, and corresponding pyroelectric coefficient increases from 2.46 to 2.81 μC/(cm^2^∙°C) at *T*_d_. For refrigeration, the indirect measurement demonstrates the Δ*T* maximum increases from 1.1 K to 1.4 K, indicating an enhanced electrocaloric effect. Moreover, the optimized energy storage effect is observed after La doping. With appearance of “slimmer” *P*-*E* loops, both calculated recoverable energy storage density *W*_rec_ and storage efficiency *η* increase to 0.23 J/cm^3^ and 22.8%, respectively. These results denote La doping conduces to the improvement of non-piezoelectric properties of BNT-based ceramics in a certain range. Therefore, La doping should be an adopted modification strategy for lead-free ceramics used in areas like refrigerator and pulse capacitors.

## 1. Introduction

Ferroelectric ceramics are a special functional material associated with various energy forms, such as electric, thermal, mechanical and so forth. Due to the significant capability of energy transformation, ferroelectric ceramics have been widely applied to electric devices, resulting in an enormous commercial market for excellent lead-based ceramics. However, many serious environmental issues arose during the long-term usage of toxic heavy metal lead so that a great deal of attention is recently devoted to exploit lead-free substitution. In view of the same valence electron arrangement of Pb^2+^ and Bi^3+^, (Bi_0.5_Na_0.5_)TiO_3_ (BNT) ceramic system was invented in the 1960s and turned out to be a promising candidate due to its high remnant polarization and strong electromechanical coupling effects [1,2]. As many modified multicomponent BNT-based ceramics came into being, although several shortcomings have been overcome, a common difficulty called depolarization phenomenon emerges that polarization, as well as piezoelectric properties, disappears at some elevated depolarization temperature *T*_d_ [3]. Massive researches reveal that *T*_d_ (often around 100 °C) is far lower than the typical curie point of lead-based ceramics (beyond 300 °C) [4]. Hence, depolarization behavior gives rise to an unwanted narrower working temperature range for piezoelectric applications.

The current prevailing perspective attributes the depolarization phenomenon to the impact of the characteristic transition of BNT-based systems from ferroelectric (FE) to ergodic relaxor (ER) state [5,6]. At room temperature, the original BNT-based ceramics generally reside in nonergodic relaxor (NR) state, where a great number of polar nanoregions (PNRs) having long correlation length disperse in matrix. Under the influence of strong external electric field, these PNRs cluster and reorient to form polar domains irreversibly, triggering the transformation from NR to FE [7]. However, when ambient temperature rises to the FE-ER transition temperature *T*_F-R_, domains will decompose into PNRs again, resulting in the degeneration of piezoelectric properties [8,9]. There is a slight distinction between *T*_d_ and *T*_F-R_, since their corresponding micro-process is not totally identical [10]. Although the FE-ER transition deteriorates domain stability, it provides an efficient method to bring a highly ordered structure to a disordered one, which is a crucial factor for many non-piezoelectric applications of BNT-based systems such as electrocaloric refrigeration and energy storage.

Electrocaloric effect (ECE) refrigeration is a new environmentally friendly solid state cooling technology. Since an appreciable ECE temperature variation Δ*T* = 12 K in Pb(Zr_0.95_Ti_0.05_)O_3_ thin films was reported by Mischenko et al. [11], the application prospect of this physical refrigeration has gradually been accepted. Later, Saranaya et al. synthesized Pb(Mg_1/3_Nb_2/3_)_0.65_Ti_0.35_O_3_ thin film, which exhibits a wonderful Δ*T* = 31 K of ECE under a very high electric field above 70 kV/mm [12]. Although thin films tend to gain more evident Δ*T*, insufficient heat capacity reminds that bulk ceramics should be taken into consideration seriously. Inasmuch as more defects decrease the electric breakdown field strength, the enhanced ECE of bulks should rationally resort to higher entropy change induced by order-disorder phase transition, rather than stronger applied electric field as performed in thin films. Under a driving electric field of 7 kV/mm, Zhang et al. studied a small amount of LiNbO_3_ modified 0.88(Na_1/2_Bi_1/2_)TiO_3_-0.12BaTiO_3_ ceramics and obtained an improved Δ*T* = 1.71 K of ECE [13]. Wang et al. incorporated (Bi_0.5_K_0.5_)TiO_3_ (BKT) into BNT ceramics to prepare an optimized component achieving Δ*T* = 1.08 K of ECE [14]. There is an obvious reduction of phase transition temperature in their experiments, implying the FE-ER transition is a considerable direction for the purpose of acquiring high ECE.

Apart from that, ferroelectric ceramics also show potential in capacitors for the sake of great dielectric constant, but the domain reorientation will bring on a substantial loss of energy storage and be detrimental to recoverable energy storage density and storage efficiency. In order to reduce the loss during circulation, ceramic capacitors always pursue a low remnant polarization. For instance, Zhang et al. introduced some SrTiO_3_ into BNT-BST-KN system, with Sr^2+^ substitution in A-site, a slender *P*-*E* hysteresis loop and an optimized energy storage density of 3.72 J/cm^3^ arose [15]. Kang et al. researched BNT-ST-FN ceramics and revealed that a slimmer *P*-*E* hysteresis loop raises recoverable energy storage density to 1.82 J/cm^3^ [16]. Several NaNbO_3_ modified BNT-based solid-solution components exhibit high energy storage efficiency as well [17]. Due to domain decomposition, samples located in the transition region between FE and ER state often display shrinking *P*-*E* loops like anti-ferroelectricity, suggesting the FE-ER transition helps to improve energy storage as well.

In summary, for non-piezoelectric applications like ECE and energy storage, the utilization of the FE-ER transition is worth noting. Although the origin of the transition is not well understood today, the prevailing theory attributes it to the random field induced by lattice distortion and ion substitution [8]. It is rational to incorporate some large and non-equivalent ions into BNT-based matrix to modify the FE-ER transition and obtain enhanced properties. Previous studies on the large strain of La-doped ceramics have confirmed that La is an effective dopant for modulating the FE-ER transition [18,19]. In this paper, the idea that utilizes the FE-ER transition to optimize non-piezoelectric performance has been inspected. Since the FE-ER transition is an intrinsic property of BNT-based ceramics [5], this strategy can be easily extended to other systems with different dopant or solid solution ingredient. Binary ceramic 0.93(Bi_0.5_Na_0.5_)TiO_3_-0.07Ba(Ti_0.945_Zr_0.055_)O_3_ (BNT-BZT) is representative in BNT-based systems with excellent piezoelectric and pyroelectric properties [20], chosen in this paper as an ideal experimental matrix. In order to modulate ergodic degree, La^3+^ ion was introduced. The influence of La dopant on the FE-ER transition and electric properties have been investigated in detail.

## 2. Materials and Methods

A series of samples 0.93(Bi_0.5_Na_0.5_)TiO_3_-0.07Ba(Ti_0.945_Zr_0.055_)O_3_-*x* mol% La (abbreviated as BNT-BZT-*x*La, *x* = 0, 0.2, 0.4, 0.6, 0.8) were synthesized by solid-state sintering method. Based on the corresponding stoichiometric formula, carbonates Na_2_CO_3_(Acros Organics 99%), BaCO_3_(Alfa Aesar 99%) and oxides Bi_2_O_3_(Alfa Aesar 99.9%), ZrO_2_(Aladdin 99%), TiO_2_(Acros Organics 99%), and La_2_O_3_(Aladdin 99.99%) were weighed. For the distinction of calcined temperature, we first prepared BZT: ball-milled mixed powders for 24 h, then calcined at 1200 °C for 2 h. Next, BZT mixed with the others, ball-milled for 24 h, calcined at 820 °C for 3 h, ball-milled again for 12 h. The dried slurry pressed into pellets under 400 MPa with binder PVA (5%), sintered at 1130 °C for 4 h in a covered alumina crucible to reduce Bi and Na evaporation. Finally, silver electrode was applied on both sides of the pellets.

The structure of the unpoled samples was determined by X-ray diffractometer (Bruker D8 Advance, Karlsruhe, Baden-Württemberg, Germany) using Cu Kα radiation for 2*θ* range from 20° to 70°. All polarization-electric field hysteresis loops were measured in silicone oil bath with a period of 1 s by Precision premier II (Radiant Tech, Los Angeles, California, USA). The surface of the samples was obtained by scanning electron microscopy (Phenom pro, Eindhoven, The Netherlands). Samples were poled by an external electric field of 5 kV/mm for 15 min at room temperature and left for one day before electrical measurements. Some apparatuses were utilized as follows: Berlincourt meter (Institute of acoustic, Chinese academic society, ZJ-6A, Beijing, China) for piezoelectric constant *d*_33_, impedance analyzer (Agilent HP4294A) for the electromechanical coupling factors *k*_p_ and *k*_t_, Precision LCR meter (Agilent E4980A) for relative permittivity and loss at discrete frequencies 1 kHz, 10 kHz, 100 kHz in a temperature range from 30 to 400 °C. To fulfill the thermally stimulated depolarization current experiment, samples were placed on a hot stage (Linkam THMS300) with accurate heating rate control, and two probes, connected to external circuit, were attached to both sample surfaces. During heating, the pyroelectric current was recorded in real time by a semiconductor measurement system (Keithley 4200SCS). A steady heating rate of 3 °C/min was chosen to measure the depolarization temperature.

## 3. Results and Discussion

**Figure 1:**
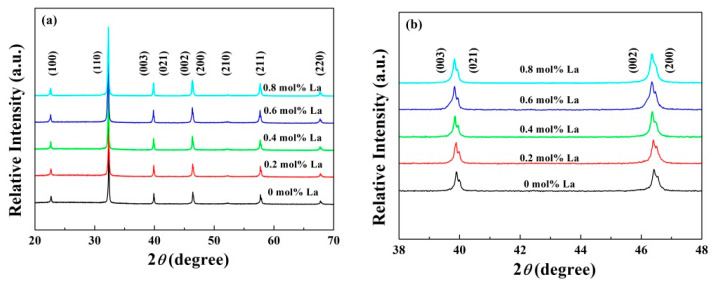
XRD patterns of BNT-BZT-*x*La ceramics in a 2*θ* range of (**a**) 20° to 70° and (**b**) 38° to 48° at room temperature.

The X-ray diffraction results (XRD) of all the ceramic components with different La contents are given in Figure 1a. Only a perovskite-type ABO_3_ structure pattern can be detected within the equipment precision, demonstrating La ions diffused in the crystal well such that no secondary impurity phase occurs. As depicted in Figure 1b, two unambiguous diffraction peaks (002) and (200), implying a tetragonal symmetry, located around 46.5°, while another peak splitting of (003) and (021), implying rhombohedral symmetry, appears around 40°. It turns out that a morphotropic phase boundary (MPB) in which rhombohedral-tetragonal symmetry coexists was formed in all the components. However, a slight tendency of peak (002) and (200) merging into peak (202) was also observed, which is in accordance with the result reported by other documents that La induces the crystal structure into pseudo-cubic phase [21]. After sintering, the valence state of La remained +3 [22]. La^3+^ ions possess a higher radius (1.36 Å) than Bi^3+^ (1.17 Å) and Na^+^ (1.18 Å), and far beyond Ti^4+^ (0.605 Å), so that they tend to substitute in A site, which is responsible for the diffraction peaks shifting to lower angles as La content increases.

**Figure 2:**
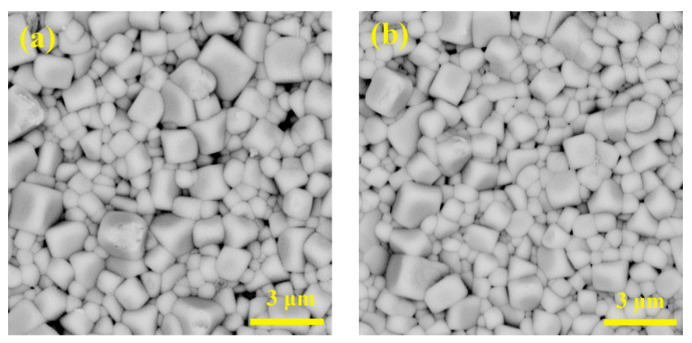

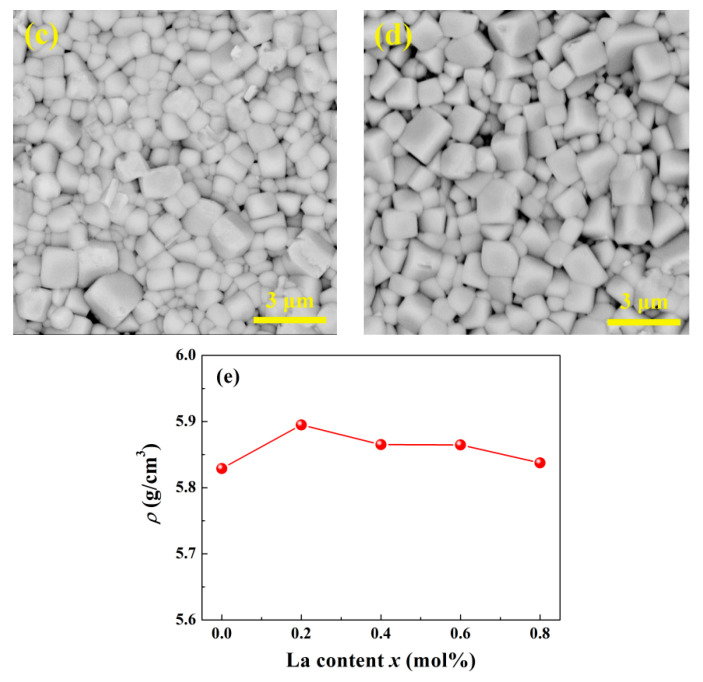
SEM micrographs of BNT-BZT-*x*La ceramics: (**a**) *x* = 0, (**b**) *x* = 0.2, (**c**) *x* = 0.4, (**d**) *x* = 0.8, (**e**) the density of sintered samples.

Figure 2 presents the surface morphology of BNT-BZT-*x*La samples. Under the influence of tetragonal BZT, ceramic grains show a cubic shape with distinct boundaries. All the samples grew sufficiently, with well-formed plump grains and few pores. The average grain size of pure BNT-BZT is about 1.33 μm. As La content increased, it decreased to 1.07 μm for the component *x* = 0.4, and then bounced to 1.36 μm for the component *x* = 0.8. The grain size of the component with higher La proportion seemed to exhibit better consistency. Resorting to Archimedes method [23], we measured density *ρ* of all the samples. As shown in Figure 2e, pure BNT-BZT possesses a high density of 5.82 g/cm^3^, demonstrating a commendable compactness [24]. With La dopant incorporated, *ρ* will slightly increase before its decline, which may be associated with the distribution of La^3+^ ions [25].

**Figure 3:**
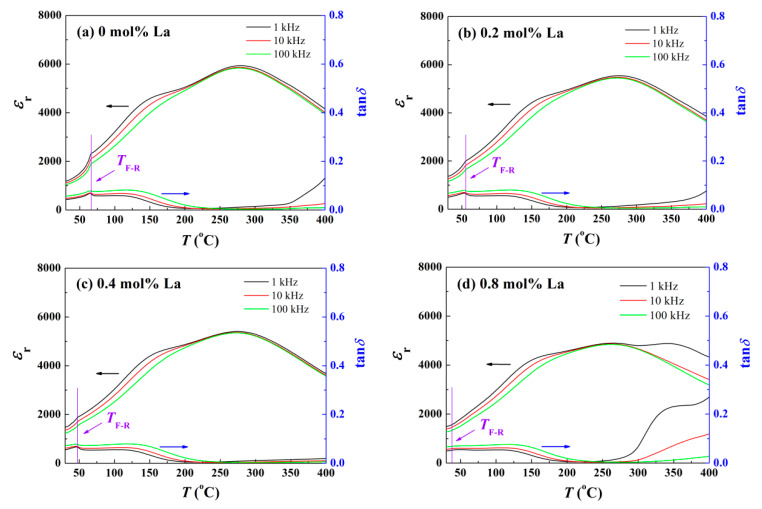
Temperature-dependent dielectric constant and loss spectra of BNT-BZT-*x*La ceramics: (**a**) *x* = 0, (**b**) *x* = 0.2, (**c**) *x* = 0.4, (**d**) *x* = 0.8.

To examine the La doping impact on ergodic degree of BNT-BZT ceramic, corresponding depolarization behavior has been investigated, and temperature *T* dependence of relative dielectric constant *ε*_r_ ranging from 30 °C to 400 °C is shown in Figure 3. All the components manifest obvious relaxation nature of BNT-BZT, such as an extensive dielectric peak at the Curie point. In contrast with the case of the unpoled samples, there exists the leftmost dielectric constant peak that represents the FE-ER transition in the *ε*_r_-*T* diagram of the poled ones. The corresponding *T*_F-R_ is marked in Figure 3. At room temperature, PNRs in the virginal NR state behave slower dynamics so that external electric field will be able to increase their correlated length and align them to form domains [8]. For strong interaction among coupling dipoles in block domain, the dipole reorientation is relatively difficult and sluggishly responds to the variation of high frequency alternating current (AC) field. As a consequence, relative dielectric constant *ε*_r_ is not sensitive to test frequencies [26]. When temperature rises to *T*_F-R_, thermal fluctuation provides sufficient energy to break the long-range order domains down into dynamic active PNRs. Due to smaller dipole cluster and lower correlated length in ER state, the polarization state of PNRs could be capable of following the variation of AC field, which brings on the obvious frequency dependence of *ε*_r_ [27]. The leftmost dielectric constant peak in the *ε*_r_-*T* diagram, as well as a loss peak in the tan*δ*-*T* curves, is formed with the change of frequency characteristics. As depicted in Figure 3a, *T*_F-R_ of pure BNT-BZT ceramic is 66.6 °C. However, with La ions incorporated, lattice distortion and local composition chaos are further induced. The resultant random field deepens ergodic degree of samples so that *T*_F-R_ decreases continuously to 38.2 °C for the component *x* = 0.8. This tendency is consistent with our prediction, which helps to return the ceramic system back to its original state.

**Figure 4:**
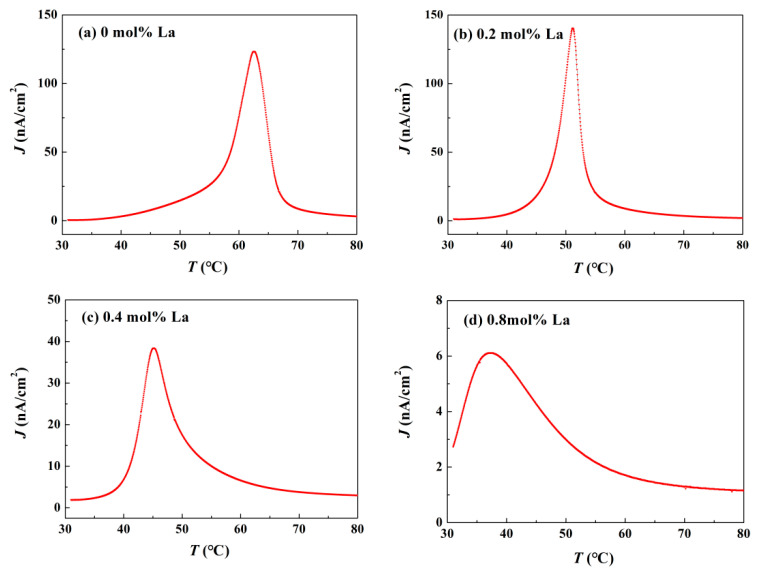
Temperature-dependent pyroelectric current density of poled BNT-BZT-*x*La ceramics: (**a**) *x* = 0, (**b**) *x* = 0.2, (**c**) *x* = 0.4, (**d**) *x* = 0.8.

As another efficient manifestation of ergodic degree variation, thermally stimulated depolarization current experiment (TSDC) provides some important information on the diminishment of polarization and domains. During steady heating process, with the system state changing from FR to ER, the bound charges are gradually neutralized so that the free charges attached to ceramic surface, which support electric neutrality after poling, are released to external circuits to form depolarization current [28]. The depolarization current density *J*-temperature *T* curves of TSDC are presented in Figure 4. Apparently, a continuous one-step depolarization behavior occurs in all the components. The temperature corresponding to depolarization current density peak is *T*_d_. As La content increases, *T*_d_ monotonously decreases from 62.6 °C to 37.2 °C, indicating an enhanced ergodic degree. Due to an appropriate modulation of La^3+^ ions on domain stability, the domains in the component *x* = 0.2 are apt to resolve simultaneously, which results in a slimmer and higher current peak. Pyroelectric coefficient *p* determined by [28]

(1) $$p=\frac{J(T)}{dT/dt}$$

increases from 2.46 μC/(cm^2^∙°C) (*x* = 0) to 2.81 μC/(cm^2^∙°C) (*x* = 0.2) at *T*_d_, which suggests La doping will be an aid to improve response of BNT-based ceramics to infrared radiation. Optimized component *x* = 0.2 possesses a superior pyroelectric effect to most other lead-based or lead-free ceramics, such as (K, Na)NbO_3_-based system or PZT system [29]. Then a rapid degression to 0.12 μC/(cm^2^∙°C) (*x* = 0.8) follows with more La incorporated. Since *T*_F-R_ of the component *x* = 0.8 approaches to room temperature, the domain stability rapidly decreases. As a consequence, a portion of unstable domains will decompose after poling electric field is removed, giving rise to a deficient remnant polarization *P*_r_. In view of the fact that pyroelectric current originates from the bound charges neutralizing [28], i.e., the reduction of *P*_r_, the dramatic fall in pyroelectric coefficient for the component *x* = 0.8 is rational. More detailed discussion will be carried out in the section on *P*-*E* loops.

**Figure 5:**
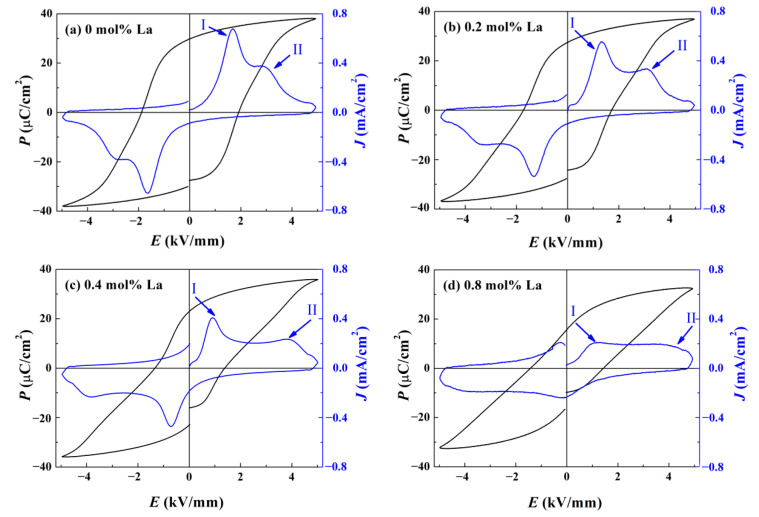
Component-dependent *P*-*E* loop and *J*-*E* curve of BNT-BZT-*x*La ceramics: (**a**) *x* = 0, (**b**) *x* = 0.2, (**c**) *x* = 0.4, (**d**) *x* = 0.8.

As a typical character of ferroelectrics, the polarization *P*-electric field *E* hysteresis loop, depicted in Figure 5, offers some basic information on domain switching and ferroelectric properties. For the component *x* = 0, *P*-*E* loop manifests a slightly constricted shape. With La content increasing, *P*-*E* loop becomes “slimmer” and “slimmer”, causing a reduced remnant polarization *P*_r_ and coercive field *E*_c_. To gain an insight into this shrinking behavior, we summarized the variation of polarization current density *J* in Figure 5 as well. Due to the deficiency of *T*_F-R_, pure BNT-BZT ceramic exhibits a certain relaxation behavior at room temperature, showing a deformed *P*-*E* loop similar to antiferroelectric materials. Accordingly, *J*-*E* curve appears with two peaks. When La^3+^ ions entered into the matrix lattice, resulting variation of random field deepened ergodic degree, and the impact should be considered under different electric loading conditions. For low loading, an enhanced ergodic degree facilitates domain destabilization and makes them switch easily. Hence, a depolarization current peak I near *E*_c_, resulting from the fact that *E*_c_ brings on drastic domain reorientation, shifts to the direction of lower electric field as more La dopant was introduced. Statistically speaking, there will be more dynamically active PNRs in the samples with higher degree of ergodicity so that a portion of energetic PNRs demand for larger loading to nucleate and grow into domains. Another peak II will be generated as these energetic PNRs transform into domains under high loading, and it moves to higher electric field as La content increases. As ergodic degree increases, the combination of these impacts deforms *P*-*E* loop further, causing an antiferroelectric loop-like shape [30]. The fatigue maybe decreases the altitude of the peaks, but their locations will be unaffected. Hence, the judgment of ergodic degree will be reliable. Although the “soft” doping effect of higher valence La^3+^ empirically leads to enhanced ferroelectricity [31], the variation of ergodic degree dominates in our research, resulting in an evident reduction of *P*_r_ and *E*_c_.

**Figure 6:**
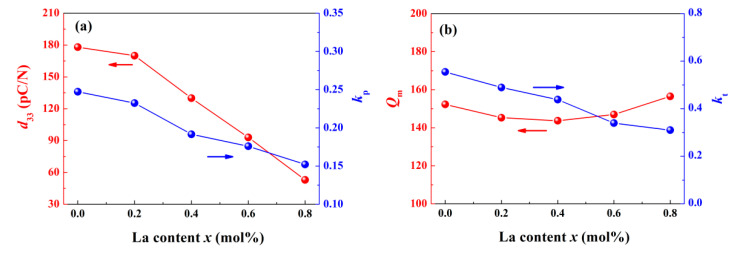
Component-dependent (**a**) *d*_33_ and *k*_p_, (**b**) *Q*_m_ and *k*_t_ of BNT-BZT-*x*La ceramics.

To further reveal the variation of piezoelectric properties, several important parameters were measured and depicted in Figure 6. Pure BNT-BZT shows superior performance to most other BNT-based binary systems [2], for instance, piezoelectric constant *d*_33_ of the component *x* = 0 reaches 178 pC/N. With increasing La content, *d*_33_ decreases to 53 pC/N for the component *x* = 0.8 as the similar tendency as planar electromechanical coupling factor *k*_p_ and thickness electromechanical coupling factor *k*_t_. For the reason of lower domain stability, one of the distinct characteristics of deepened ergodic degree is continuous deterioration of piezoelectric behavior [8]. The phenomena can be regarded as the consequence of *P*_r_ reduction. For the same reason, as presented above in Figure 4, the altitude of depolarization current density peak declines in the samples with larger La content.

**Figure 7:**
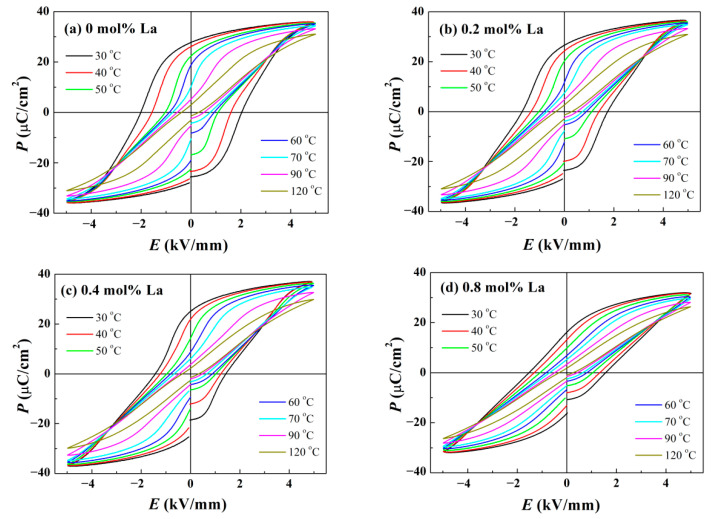
Temperature-dependent *P*-*E* hysteresis loops of BNT-BZT-*x*La ceramics: (**a**) *x* = 0, (**b**) *x* = 0.2, (**c**) *x* = 0.4, (**d**) *x* = 0.8.

**Figure 8:**
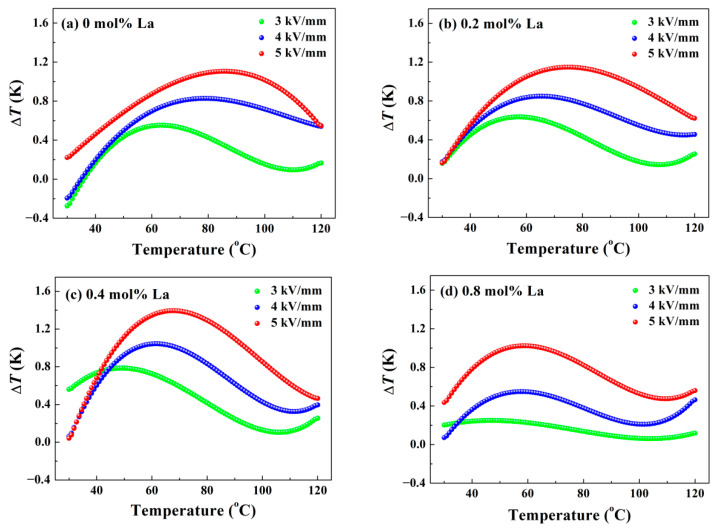
ECE temperature change Δ*T* in adiabatic process as a function of test temperature: (**a**) *x* = 0, (**b**) *x* = 0.2, (**c**) *x* = 0.4, (**d**) *x* = 0.8.

**Figure 9:**
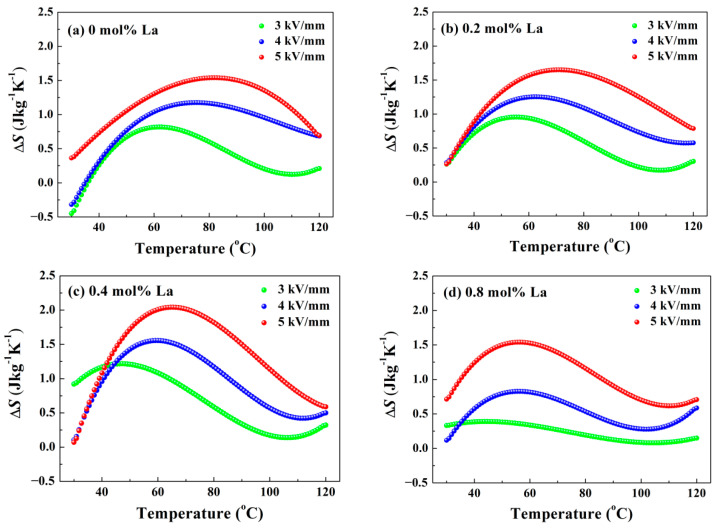
ECE entropy change Δ*S* in isothermal process as a function of test temperature: (**a**) *x* = 0, (**b**) *x* = 0.2, (**c**) *x* = 0.4, (**d**) *x* = 0.8.

To calculate ECE by the indirect measurement, the temperature-dependent *P*-*E* hysteresis loops have been investigated firstly, which are provided in Figure 7. When temperature is rising close to *T*_F-R_, *P*_r_ will undergo an evident reduction from which a great entropy change originates. The mode of ECE refrigeration resembles Carnot’s cycle of heat engine [32], including two adiabatic and two isothermal processes. Customarily, the intensity of the ECE is quantitatively denoted by the temperature change Δ*T* in adiabatic process [33]:

(2) $$\Delta T=-\frac{1}{\rho }\int_{E1}^{E2}\frac{T}{C_{E}}{\left(\frac{\partial P}{\partial T}\right)}_{E}dE$$

or by the entropy change Δ*S* in isothermal process [33]:

(3) $$\Delta S=-\frac{1}{\rho }\int_{E1}^{E2}{\left(\frac{\partial P}{\partial T}\right)}_{E}dE$$

where *ρ* is the density of samples offered in Figure 2e, *C*_E_ is the specific heat at constant electric field (obtained from literature [34]), and (∂*P*/∂*T*)*_E_* represents the variation rate of polarization versus test temperature under constant electric field. Since only the *P*-*E* loops at limited temperatures have been measured, (∂*P*/∂*T*)*_E_* should be obtained by derivation of *P*-*T* polynomial fitting curves. The results have been depicted in Figure 8 and Figure 9. For intuitive comparison, the conventional unit of temperature centigrade was chosen for horizontal axis. Obviously, higher electric field moves the location of the Δ*T* maximum towards high temperature, because the decomposition of domains under strong loading requires much more energy contributed by thermal fluctuation. For the same reason, the test temperature corresponding to the peaks in Figure 8 and Figure 9 is much larger than *T*_d_ and *T*_F-R_ determined in Figure 3 and Figure 4, respectively. Since ceramic possesses a higher order structure at stronger electric field, the degree of order-disordered transition will be greater. Hence, the altitude of peak dramatically increases with strong loading applied, as shown in Figure 8 and Figure 9. In order to assess ECE, the curves of 5 kV/mm were taken for example. Figure 8a reveals that pure BNT-BZT possesses a Δ*T* maximum of 1.1 K located at 85.5 °C. For La doped components, deepened ergodic degree gives rise to greater order-disordered change, so that Δ*S* increases at low temperature, as presented in Figure 9. As the conjugate variable of *S*, Δ*T* will get an increment at low temperature as well. Restricted by integral part of the calculation, this increment is ambiguous until about 40 °C. On the other hand, in accordance with the result of Figure 4, modest La content seems to concentrate the decomposition of domains, the Δ*T* and Δ*S* maximum rise to 1.4 K and 2.04 Jkg^−^^1^K^−^^1^ (*x* = 0.4), respectively. However, further La dopant addition will vitiate the ordered structure generation under high electric field, so that they decline to 1.0 K and 1.54 Jkg^−^^1^K^−^^1^ (*x* = 0.8), respectively. The optimized maximum electrocaloric coefficient Δ*T*/Δ*E* reaches to 0.28 Kmm/kV. The ECE result of the component *x* = 0.4 is better than some other BNT-based systems, such as BNT-BT and BNT-BKT [14,35]. In view of the low driving electric field of 5 kV/mm, enhanced ECE should be expected under higher loading. These variations demonstrate La doping is capable of modulating the temperature where the Δ*T* maximum appears and enhancing ECE.

**Figure 10:**
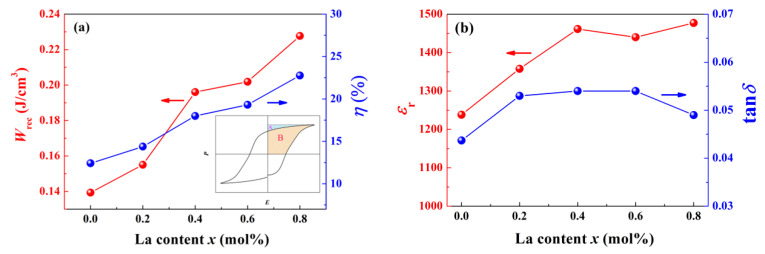
Component dependence of (**a**) *W*_rec_ and *η*, (**b**) *ε*_r_ and tan*δ* at RT (the inset in (**a**) manifests the area corresponding to recoverable energy storage density and energy loss).

The last section is devoted to energy storage, which is mostly a concern in capacitors. It is well known that ferroelectrics possess excellent dielectric constant. Hence, ferroelectric ceramic capacitors should have superior capability of energy storage [36]. In fact, for the sake of polarization hysteresis, ceramics fail to behave as paraelectrics, which release entire polarization energy after unloading, but rather a part of electric energy provided by a power source must be afforded to irreversible domain reorientation—that is, energy loss. The magnitude of this loss can be predicted by the area A enclosed by the *P*-*E* loop. By contrast, the real available recoverable energy storage density *W*_rec_ during circulation is represented by the area B between vertical axis and upper branch of the *P*-*E* loop in the first quadrant. Both A and B region are labeled in the inset of Figure 10a. *W*_rec_ can be calculated by [37]

(4) $$W_{\mathrm{rec}}=\int_{P_{r}}^{P_{\max}}E\;dP$$

To better describe the utilization of external electric field energy, energy storage efficiency *η* is defined as [37]

(5) $$\eta \;=\;\frac{W_{\mathrm{rec}}}{W}=\frac{W_{\mathrm{rec}}}{W_{\mathrm{rec}}+W_{\mathrm{loss}}}$$

To improve energy storage performance, an enhanced A/B ratio will be necessary. It is a natural thought to improve A/B ratio by slenderizing *P*-*E* loop. La doping deepens ergodic degree and shrinks *P*-*E* loop so that optimized energy storage behavior should be expected with proper La content incorporated. According to the results of Figure 5, we have calculated *W*_rec_ and *η* shown in Figure 10a. With the variation of ergodic degree, *W*_rec_ increases from 0.14 to 0.23 J/cm^3^, while energy loss monotonously decreases, so that *η* increases from 12.4% to 22.8%. Apart from this, dielectric constant remains ascending with more La introduced. These results demonstrate that the domain decomposition is beneficial to release polarization energy. Although *W*_rec_ and *η* are inferior to other studies [38,39], taking account of a low test electric field of 5 kV/mm and the fact that *T*_F-R_ is still above room temperature, the best effect of La doping seems to not be reached. However, these results support the idea that utilizes the FE-ER transition to optimize non-piezoelectric performance, which should be a feasible method for acquiring superior energy storage in BNT-based ceramics.

## 4. Conclusions

In this paper, the impact of La doping on some non-piezoelectric electric properties of 0.93(Bi_0.5_Na_0.5_)TiO_3_-0.07Ba(Ti_0.945_Zr_0.055_)O_3_ (BNT-BZT) ceramics has been investigated in detail. Both the temperature dependence of relative dielectric constant and *P*-*E* loop results confirm that La doping helps to enhance ergodic degree of BNT-BZT ceramics. Although domain destabilization deteriorates the potential for piezoelectric applications, a series of non-piezoelectric properties are improved. When proper La dopant was incorporated, the domain decomposition tends to concentrate and an optimized pyroelectric coefficient *p* of the component *x* = 0.2 rises to 2.81 μC/(cm^2^∙°C) at *T*_d_. Since deepened ergodic degree leads to larger entropy variation, for ECE refrigeration, the maximum of Δ*T* increases from 1.1 K of pure BNT-BZT to 1.4 K of the component *x* = 0.4. For energy storage applications, the samples with deepened ergodic degree release stored polarization energy easier. As a consequence, both *W*_rec_ and *η* increase to 0.23 J/cm^3^ and 22.8%, respectively. Through controlling La content, the temperature at which the maximum of the above parameters appears can be modified. All these results demonstrate the feasibility of utilizing the FE-ER transition to optimize non-piezoelectric performance. La element should be a promising candidate to improve non-piezoelectric performance of ferroelectric ceramics.

## Data Availability

Data sharing not applicable.
